# Multicentre randomized controlled trial comparing ferric(III)carboxymaltose infusion with oral iron supplementation in the treatment of preoperative anaemia in colorectal cancer patients

**DOI:** 10.1186/s12893-015-0065-6

**Published:** 2015-06-28

**Authors:** W. A. A. Borstlap, C. J. Buskens, K. M. A. J. Tytgat, J. B. Tuynman, E. C. J. Consten, R. C. Tolboom, G. Heuff, N. van Geloven, B. A. van Wagensveld, C. A. C.A. Wientjes, M. F. Gerhards, S. M. M. de Castro, J. Jansen, A. W. H. van der Ven, E. van der Zaag, J. M. Omloo, H. L. van Westreenen, D. C. Winter, R. P. Kennelly, M. G. W. Dijkgraaf, P. J. Tanis, W. A. Bemelman

**Affiliations:** Department of Surgery, Academic Medical Centre, University of Amsterdam, Amsterdam, The Netherlands; Department of Gastroenterology, Academic Medical Centre, University of Amsterdam, Amsterdam, The Netherlands; Clinical Research Unit, Academic Medical Centre, University of Amsterdam, Amsterdam, The Netherlands; Department of Surgery, VU University Medical Centre, Amsterdam, The Netherlands; Department of Surgery, Meander Medical Centre, Amersfoort, The Netherlands; Department of Surgery, Spaarne Hospital, Hoofddorp, The Netherlands; Department of Surgery, Tergooi Hospital, Hilversum, The Netherlands; Department of Surgery, Sint Lucas Andreas Hospital, Amsterdam, The Netherlands; Department of Gastroenterology, Sint Lucas Andreas Hospital, Amsterdam, The Netherlands; Department of Surgery, Onze Lieve Vrouwe Gasthuis, Amsterdam, The Netherlands; Department of Gastroenterology, Onze Lieve Vrouwe Gasthuis, Amsterdam, The Netherlands; Department of Surgery, Flevo Hospital, Almere, The Netherlands; Department of Surgery, Gelre Hospital, Apeldoorn, The Netherlands; Department of Surgery, Isala Hospital, Zwolle, The Netherlands; Department of Surgery, St. Vincent’s Hospital, Dublin, Ireland

**Keywords:** Colorectal cancer, Iron deficiency anaemia, Iron supplementation

## Abstract

**Background:**

At least a third of patients with a colorectal carcinoma who are candidate for surgery, are anaemic preoperatively. Preoperative anaemia is associated with increased morbidity and mortality. In general practice, little attention is paid to these anaemic patients. Some will have oral iron prescribed others not. The waiting period prior to elective colorectal surgery could be used to optimize a patients’ physiological status. The aim of this study is to determine the efficacy of preoperative intravenous iron supplementation in comparison with the standard preoperative oral supplementation in anaemic patients with colorectal cancer.

**Methods/Design:**

In this multicentre randomized controlled trial, patients with an M0-staged colorectal carcinoma who are scheduled for curative resection and with a proven iron deficiency anaemia are eligible for inclusion. Main exclusion criteria are palliative surgery, metastatic disease, neoadjuvant chemoradiotherapy (5 × 5 Gy = no exclusion) and the use of Recombinant Human Erythropoietin within three months before inclusion or a blood transfusion within a month before inclusion. Primary endpoint is the percentage of patients that achieve normalisation of the haemoglobin level between the start of the treatment and the day of admission for surgery. This study is a superiority trial, hypothesizing a greater proportion of patients achieving the primary endpoint in favour of iron infusion compared to oral supplementation. A total of 198 patients will be randomized to either ferric(III)carboxymaltose infusion in the intervention arm or ferrofumarate in the control arm. This study will be performed in ten centres nationwide and one centre in Ireland.

**Discussion:**

This is the first randomized controlled trial to determine the efficacy of preoperative iron supplementation in exclusively anaemic patients with a colorectal carcinoma. Our trial hypotheses a more profound haemoglobin increase with intravenous iron which may contribute to a superior optimisation of the patient’s condition and possibly a decrease in postoperative morbidity.

**Trial registration:**

ClincalTrials.gov: NCT02243735.

## Background

Colorectal carcinoma has a peak incidence in the seventh decade of life and patients often presents with comorbid iron deficiency anaemia. At least a third of patients with a colorectal carcinoma who undergo surgery, are anaemic preoperatively [1].

**Figure 1:**
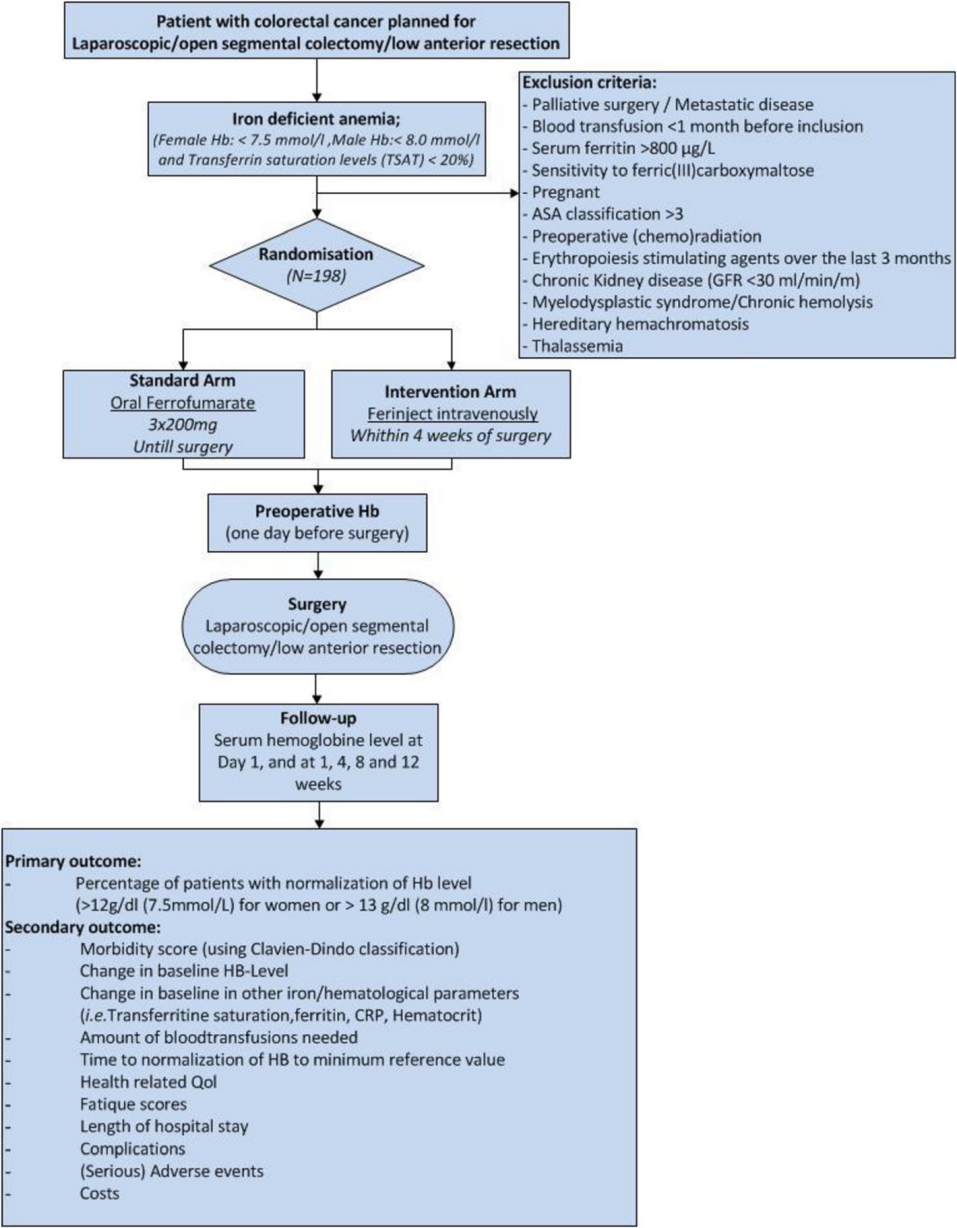
Flowchart of FIT-trial

**Figure 2:**
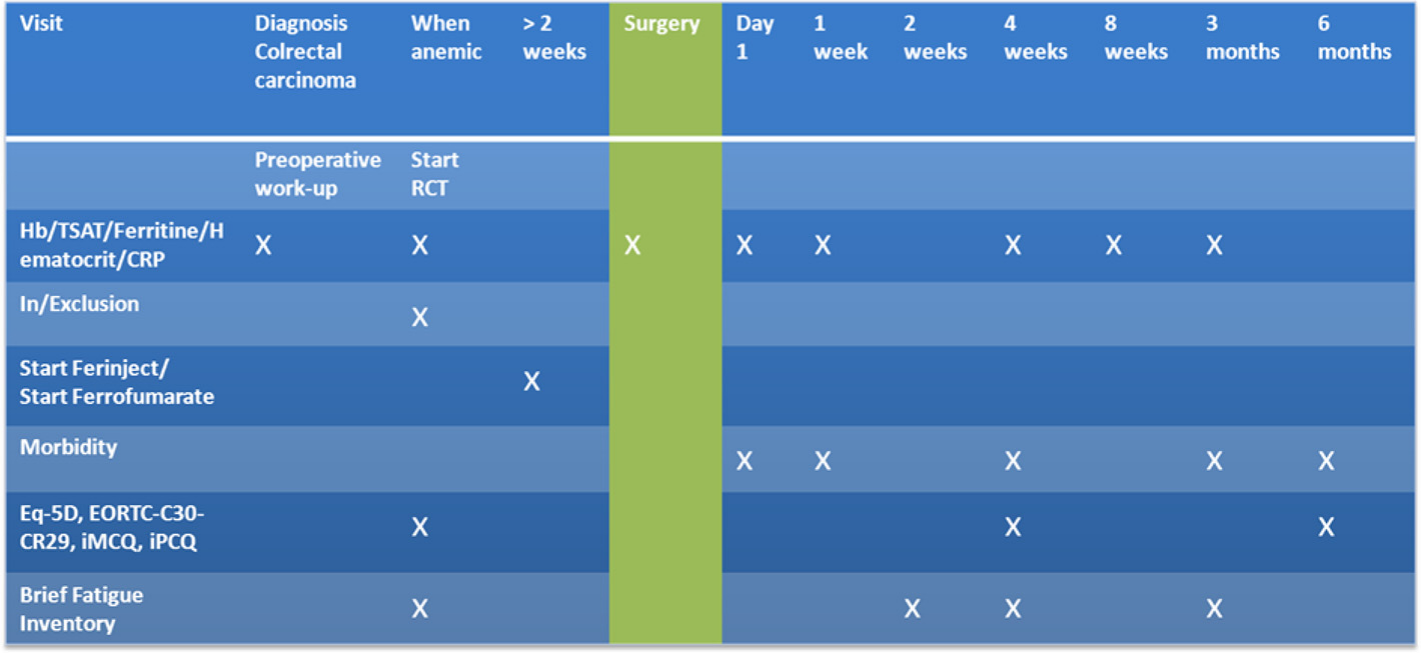
Study follow-up

Anaemia in patients with colorectal carcinoma is partly caused by gastro-intestinal (GI) blood loss and is partly cancer-related. Cancer related anaemia is multifactorial and is caused by impaired iron absorption, nutritional deficiency and anaemia of chronic disease, which is a cytokine-mediated disorder. These effects cause functional iron deficiency, which is characterized by insufficient available iron at the site of erythroblast production (iron restricted erythropoiesis) with adequate iron stores [2]. In addition, iron is an essential component of a large number of human metabolic enzymes, including ribonucleotide reductase and NADH dehydrogenase [3, 4]. Therefore iron deficiency even without concomitant anaemia is associated with fatigue [5, 6], impaired physical performance and cognitive function [7–9].

Currently there are three options in the treatment of anaemia: blood transfusion, erythropoietin stimulating agents (ESA) and iron supplementation. Blood transfusion and ESA are effective modalities in increasing haemoglobin (HB) levels, however both modalities should be given with caution in oncologic patients as they are associated with an increased risk of cancer recurrence [10, 11] and ESA is even associated with an 17 % increase in overall mortality in oncologic patients [12].

With the application of laparoscopic surgery, blood loss is limited and blood transfusions are rarely necessary. In the LAFA study including 50 % open and 50 % laparoscopic segmental colectomies the blood transfusion rate was only 4 %[1]. This decreasing rate of blood transfusion could be a reason for the moderate attention for the treatment of light to moderate anaemia in the preoperative setting. However it has been shown that preoperative anaemia, even to a mild degree, is independently associated with an increased risk of morbidity and 30-day mortality [12, 13].

There is no standard of care in the treatment of light to moderate anaemia in the preoperative setting; some will have oral iron prescribed others not. A more profound treatment of iron deficiency anaemia could play a crucial role in optimizing patient’s condition prior to surgery [5]. Even tumour response on chemotherapy, as suggested by Lindsey [14], could be negatively influenced by low HB levels [15, 16]. In the Netherlands, average waiting time before surgery in case of a colorectal carcinoma is two to three weeks. This period could be used more effective in the optimisation of patients towards surgery. Studies on iron supplementation (both oral and intravenous) prior to orthopaedic [17] and gynaecologic surgery [18] showed that iron supplementation is effective in treating anaemia, reducing blood transfusions and also reducing length of stay [19].

However, in patients with colorectal carcinoma and concomitant anaemia current evidence on the role of iron supplementation seems inconclusive. This is due to methodological short comings of the studies, with small study populations [20, 21], heterogeneity in iron preparations supplied, the lack of data on surgical outcomes and most importantly due to inclusion of both anaemic and non-anaemic patients [20, 22]. All of the above underlines the need for a new trial on the efficacy of pre-operative iron therapy in treatment of anaemia in patients with a colorectal carcinoma and its effect on outcomes after surgery.

## Methods/design

The aim of this multicentre trial is to investigate which route of iron supplementation is superior in the treatment of iron deficiency anaemia in patients with colorectal carcinoma. By enhancing the preoperative condition of the patient, this trial aims to optimize postoperative outcome in anaemic patients. It is our hypothesis that a more profound approach of anaemia with intravenous iron will lead to a higher percentage of patients with normalization of Hb-level (>12 g/dl (7.5 mmol/l) for women and > 13 g/dl (8 mmol/l) for men) undergoing surgery, which potentially reduces morbidity, length of hospital stay, improves quality of life, decreases fatigue and could be more cost effective compared to current practice with oral substitution of iron.

In addition, an economic evaluation of intravenous iron versus oral iron will be performed. The evaluation will be performed from a societal perspective as (i) a cost-effectiveness analysis with the costs per responder to iron supplementation therapy as primary outcome and (ii) a cost-utility analysis with the costs per quality adjusted life-year (QALY) as primary outcome. The cost effectiveness analysis closely relates to clinical efficacy measure and allows for setting priorities in treatment of anaemia in colorectal cancer patients. The cost-utility analysis allows for a comparison of the societal impact of intravenous iron supplementation on post-operative recovery, such as improved health utility, shorter length of stay and earlier return to daily activities.

### The primary aim of the FIT trial

1. To compare the percentage of patients with normalization of Hb-level (>12 g/dl (7.5 mmol/l) for women and > 13 g/dl (8 mmol/l) for men) after intravenous versus oral iron therapy in patients undergoing curative surgery for colorectal carcinoma.

Secondary aims of the FIT trial are:

2) To analyse the effect of preoperative iron therapy (intravenous versus oral) on postoperative morbidity, length of stay, amount of blood transfusions needed and quality of life and fatigue scores.
3) To determine the cost effectiveness of preoperative intravenous iron substitution in comparison with oral substitution.

### Study design

The study is designed as a multicentre randomized clinical trial comparing ferric(III)carboxymaltose infusion with oral supplementation of ferrofumarate in the treatment of preoperative anaemia in colorectal cancer patients. Patients with a proven iron deficiency anaemia who undergo segmental colonic resection or (low) anterior resection because of M0-stage colorectal carcinoma are eligible for inclusion. Patients undergoing local excision of a rectal carcinoma (TEM/EMR/ESR) will not be included. In Fig. 1 you can find the flowchart of the FIT-trial.

When a patient has got a proven iron deficiency anaemia and the patient does not meet the criteria for exclusion, he/she will be included in the trial. Written informed consent will be obtained from each patient. The patients’ albumin and C-reactive protein (CRP) levels are used to derive a Glasgow Prognostic Score (GPS) which can be used as a predictor of post-operative outcome.

Patients will be computer randomized in random blocks of sizes 2 or 4 for either intravenous- or oral iron. Randomization will be stratified for age, colon or rectal carcinoma, open or laparoscopic operation and baseline Hb (8–10 g/dl vs 10–13 g/dl).

Apart from routine 5 year oncological follow-up, patients will be followed more intensively until 6 months after surgery as part of this trial. Hb, transferrin saturation (TSAT), ferritin, Hematocrit and CRP levels are measured at postoperative day 1, day 7, at 4, 8 and 12 weeks to monitor the anaemia.The follow-up schedule is detailed in Fig. 2.

### Study procedures

Surgical procedures include all laparoscopic and open segmental resections. Patients randomized to intravenous iron will receive ferric(III)carboxymaltose dosed according to Summary of Product Characteristics (SPC) depending on body weight and Hb-level and it will be administered in one or two infusions with one week in between, prior to the operation. (also see Table 1). The maximum dose administered to the patient may not exceed 15 mg/kg and a maximum of 1000 mg may be administered per week. When the patient has a weight of lower than 35 kg, a dose of 500 mg will be given. Patients randomized to intravenous iron will receive iron infusion on the short stay/colon care unit. Ferric(III) carboxymaltose has to be infused in a period of 15 min. Patients randomized to standard care with ferrofumarate will receive three tablets of 200 mg daily from randomisation until day before surgery. This dose is conform national farmacotherapeutic protocol [23]. When patients remain anaemic postoperatively, they will be supplied with iron according to the allocated study arm.

**Table 1:** Determining the cumulative dose of ferric(III)carboxymaltose

Hb mmol/L (g/dl)	Patients weight: 35–70 kg	Patients weight: > 70 kg
<6.2 mmol/L (10 g/dl)	1500 mg	2000 mg
>6.2 mmol/L (10 g/dl)	1000 mg	1500 mg

N.B: The maximum dose administered per week is 1000 mg. The maximum dose administered per patient may not exceed 15 mg/kg. Therefore, for patients with a weight under 67 Kg, all calculated doses should be given in two infusions (as 1000/15 = 66.7)

### Study population

The patient population consists of patients with M0-stage colorectal carcinoma and concomitant iron deficiency anaemia (Hb <7,5 mmol/l (12 g/dl) for women and Hb < 8 mmol/l (13 g/dl) for men and TSAT < 20 %) who will undergo a laparoscopic/open segmental colonic resection or (low) anterior resection. Additionally the patient should be 18 years of age or older and informed consent must be obtained. Exclusion criteria are: palliative surgery/metastasized disease, blood transfusion within one month before screening, serum ferritin above 800 μg/L, pregnancy, contraindication to use ferric(III)carboxymaltose or ferrofumarate, ASA classification higher than 3, the use of erythropoietin stimulating agents within three months before screening, chronic kidney disease (Glomerular filtration rate (GFR) <30 ml/min/m), myelodysplastic syndrome, elevated liver enzymens (more than three times normal value), hereditary hemochromatosis, thalassemia, haemolytic anaemia/ chronic haemolysis.

### Outcome parameters

Our primary endpoint is the percentage of patients with normalization of Hb-level from start treatment until surgery (Hb >12 g/dl (7.5 mmol/L) for women and Hb >13 g/dl (8.0 mmol/L) for men). Our secondary endpoints are morbidity, assessed with the Comprehensive Complication index, amount of blood transfusions needed, length of stay, absolute change in HB from baseline prior to surgery and postoperatively, time needed to achieve normalization of Hb-level, change in baseline of other iron/haematological parameters (TSAT, ferritin, CRP), health-related quality of life and fatigue scores (EQ-5D, EORTC-C30, EORTC -CR29 iMCQ, BFI & iPCQ), relation between anaemia and Glasgow Prognostic Score (derived from CRP and Albumin) and cost-effectiveness of intravenous iron treatment compared to oral. Blood samples to assess Hb- and iron values will be taken at baseline, at admission, postoperative day 1, 7 and after 1-,2- and 3 months.

### Sample size calculation

The principal analysis will be an intention-to-treat comparison of the proportions of patients with iron deficiency anaemia between the two study groups. The trial is designed as a superiority trial, hypothesizing a greater percentage of patients achieving normalization of Hb-level (called ‘responder’) in favour of infusion of ferric(III)carboxymaltose compared to oral iron suppletion. Our power calculation is based on the study of Seid et al. [24], which compared ferric(III)carboxymaltose with oral ferrous sulphate in a population of post-partum women with an iron deficiency anaemia. The proportion achieving a normalization of Hb after two weeks of treatment was 55 % in the intravenous iron group and 35 % in the oral iron group. We expect that the efficacy of the iron therapy is lower in patients with a colorectal carcinoma. Therefore, the expected percentage of patients who achieve normalization of Hb-level (Hb >7.5 mmol/l (12 g/dl) for women and Hb >8.0 mmol/l (13 g/dl) for men) is 45 % in the intravenous iron group and 25 % in the oral iron group. Based on these proportions, a sample size of 89 patients per group is needed for a Chi square test to achieve 80 % power at a two sided alpha of 0.05. With an estimated loss to follow up of 10 %, a sample size of 198 is calculated. We used nQuery advisor version 7.0 to calculate the sample size.

### Data-analysis

The intention-to-treat population will include all patients who give their informed consent, for whom there is confirmation of successful allocation of a randomization number, and who received at least one dose of the study medication. Statistical analyses will be performed using SPSS software for Windows version 19.

The primary endpoint, the percentage of patients with a normalised Hb-level at admission will be compared between the two study groups on a intention to treat basis. Using a two-sided Chi-square test at a significance level of 0.05. All data will be collected in an electronic database. The outcome parameters will be analysed with appropriate statistical tests by a statistician blinded for the treatment allocation using the statistical program SPSS software for Windows version 19.

Appropriate summary descriptive statistics will be determined for all secondary endpoints at each visit using raw scores. To assess morbidity the continuous scale of the Comprehensive Complication Index [25] and the categorical Clavien-Dindo classification will be used, for which appropriate statistical tests will be undertaken (the Mann Whitney U test, in case of non-normal distribution, and the Chi squared test respectively). For continuous secondary endpoints, either analysis of variance or analysis of covariance models will be used and where necessary, the repeated measures procedure will be implemented.

Quality of life data (e.g. EORTC-C30, EORTC-CR29 and EQ-5D) will be graphically represented across all time points and analysed using a repeated measures analysis of variance. All tests based on proportions will be analysed using a logistic regression model with treatment as a factor and, where appropriate, other specified covariates to include baseline score. Time-to-event will be analysed with use of Kaplan-Meier Survival analysis and compared using the log-rank test. Where appropriate, a stratified log-rank test and Cox proportional hazard model will be used to explore the potential influences of baseline HB and other specified covariates.

The secondary efficacy analysis will be based on the Full Analysis Set (FAS) and the per protocol populations. Significance level is set at an alpha of 0.05 and no adjustment will be made for testing multiple secondary outcomes. Some significant findings are expected to occur by chance so undue consideration will not be given to any particular significant difference. Moreover, interpretation of the results will be based on patterns of differences and in conjunction with the results of the primary analyses.

### Cost analysis

Unit costing of distinct health care resources will be in accordance with national guidelines [26]. Cost data will be derived as the product sum of health care volume data and their respective unit cost. Observed health utilities based on the EQ-5D health status profiles will be linked to the lengths of the periods in between measurements to derive QALYs. Incremental cost-effectiveness and cost-utility analyses will be performed to calculate the extra cost per additional ‘responder’ and the extra costs per additional quality adjusted life year respectively. Differences between groups will be assessed by calculating 95 % confidence intervals for the mean differences after non-parametric bootstrapping, drawing at least 1,000 samples of the same size as the original sample separately for each group and with replacement. A cost-effectiveness acceptability curve will be drawn to show the probability of intravenous iron supplementation being cost- effective at willingness-to-pay values up to €80,000 per QALY. Single and multi-way sensitivity analyses will be performed to study the robustness of these findings to plausible changes in key unit costs and to alternative health utility scoring algorithms [27]. With a time horizon of six months of follow-up, no discounting of efficacy and cost data will be applied to account for time preference.

### Ethics and safety

This trial will be conducted according to the principles of the declaration of Helsinki (Fortaleza, October 2013 ) and in accordance with the Medical Research Involving Human Subjects Act (WMO) and other European guidelines, regulations and acts. Data management, monitoring and reporting of the study will be carried out in accordance with the ICH GCP guidelines. The medical ethical committee of the Academic Medical Centre, Amsterdam, the Netherlands, has approved the study protocol (NL50013.018.14). As this trial concerns the effectiveness of a medication a Data Safety Monitoring Board (DSMB) will be assigned. The DSMB will guard the safety of the included patients, give advice on continuation of the study upon superiority of one of the types of treatment, and will guard the methodological quality of the study.

## Discussion

This is the first randomized controlled trial to determine the efficacy of preoperative iron supplementation in exclusively anaemic patients with a colorectal carcinoma. In current literature there are two randomized trials on preoperative iron supplementation for colorectal carcinoma patients [20, 21]. Both included non-anaemic patients, were small sized and used a fixed dosage of iron supplementation [20]. As iron-therapy is unlikely to be effective when the patient has no deficiency this is a huge confounder in both studies. Apart from these two randomized controlled trials there are four cohort studies addressing preoperative iron supplementation in patients undergoing surgery for colorectal carcinoma [22, 28–30]. These four cohort series all focussed mainly on perioperative blood transfusion rate. Currently, in centralized centres the blood transfusion rate after colorectal surgery is around 4 % [1]. This low blood transfusion rate adds to the debate whether postoperative transfusion is the most important clinical endpoint assessing patients with anaemia.

It is well-known that anaemia is associated with impaired physical performance and lower quality of life [5]. In a retrospective cohort study of 227425 patients undergoing non-cardiac surgery Musallam et al. [13] showed that anaemia is associated with a higher postoperative morbidity. A reduction in morbidity was seen in the non-anaemic patients compared to anaemic patients from 28 % to 5 % in non-anaemic patients. A reduction in postoperative morbidity was also seen even in mild to moderate anaemic patients, compared to more severe anaemic patients. This strengthens our hypothesis that more effective treatment of anaemia improves the morbidity after surgery.

In current practice, the period between diagnosis and surgery is approximately two to three weeks. This provides a window of opportunity for treatment with iron supplementation, as it takes time to increase the Hb-level with iron therapy. Blood transfusions or erythropoietin stimulating agents (ESA) are both successful in the treatment of anaemia, but as these modalities significantly increase the risk of recurrence and even mortality, they should be given with restraint [31]. Both intravenous and oral therapy are currently used and accepted as treatment of iron deficiency anaemia. However, a study directly comparing the effectiveness of both treatment options and simultaneously assessing the correlation with postoperative outcome has never been performed. Therefore the primary aim of this trial is to provide evidence on the safest and most effective treatment option of anaemia in the limited time period of the preoperative setting.

## Abbreviations

FIT: Ferric(III)carboxymaltose infusion with oral iron supplementation in the treatment of preoperative anaemia
GI: Gastro-intestinal
ESA: Erythropoietin stimulating agents
HB: Haemoglobin
TEM: Transanal Endoscopic Microsurgery
ESD: Endoscopic Submucosal Dissection
EMR: Endoscopic Mucosal Resection
ERAS: Enhanced recovery after surgery
QALY: Quality adjusted life years
GPS: Glasgow Prognostic Score
CRP: C-reactive protein
TSAT: Transferrin saturation
SPC: Summary of Product Characteristics
GFR: Glomerular Filtration rate
FAS: Full analysis Set
