# Erratum to: Multicentre randomized controlled trial comparing ferric(III) carboxymaltose infusion with oral iron supplementation in the treatment of preoperative anaemia in colorectal cancer patients

**DOI:** 10.1186/s12893-015-0090-5

**Published:** 2015-10-08

**Authors:** WAA Borstlap, CJ Buskens, KMAJ Tytgat, JB Tuynman, ECJ Consten, RC Tolboom, G. Heuff, AAW van Geloven, BA van Wagensveld, CACA Wientjes, MF Gerhards, SMM de Castro, J. Jansen, AWH van der Ven, E. van der Zaag, JM Omloo, HL van Westreenen, DC Winter, RP Kennelly, MGW Dijkgraaf, PJ Tanis, WA Bemelman

**Affiliations:** Department of Surgery, Academic Medical Centre, University of Amsterdam, Amsterdam, The Netherlands; Department of Gastroenterology, Academic Medical Centre, University of Amsterdam, Amsterdam, The Netherlands; Clinical Research Unit, Academic Medical Centre, University of Amsterdam, Amsterdam, The Netherlands; Department of Surgery, VU University Medical Centre, Amsterdam, The Netherlands; Department of Surgery, Meander Medical Centre, Amersfoort, The Netherlands; Department of Surgery, Borstlap et al. BMC Surgery (2015) 15:78 Spaarne Hospital, Hoofddorp, The Netherlands; Department of Surgery, Tergooi Hospital, Hilversum, The Netherlands; Department of Surgery, Sint Lucas Andreas Hospital, Amsterdam, The Netherlands; Department of Gastroenterology, Sint Lucas Andreas Hospital, Amsterdam, The Netherlands; Department of Surgery, Onze Lieve Vrouwe Gasthuis, Amsterdam, The Netherlands; Department of Gastroenterology, Onze Lieve Vrouwe Gasthuis, Amsterdam, The Netherlands; Department of Surgery, Flevo Hospital, Almere, The Netherlands; Department of Surgery, Gelre Hospital, Apeldoorn, The Netherlands; Department of Surgery, Isala Hospital, Zwolle, The Netherlands; Department of Surgery, St. Vincent’s Hospital, Dublin, Ireland

Erratum to: BMC Surgery doi:10.1186/s12893-015-0065-6

Following publication of the original version of this article in *BMC Surgery* [1] it was brought to our attention that the name of one author was spelled incorrectly. The name of author A.A.W. van Geloven was incorrectly spelled as ‘N. van Geloven’.
